# Poor Prognosis with *In Vitro* Fertilization in Indian Women Compared to Caucasian Women Despite Similar Embryo Quality

**DOI:** 10.1371/journal.pone.0007599

**Published:** 2009-10-26

**Authors:** Lora K. Shahine, Julie D. Lamb, Ruth B. Lathi, Amin A. Milki, Elizabeth Langen, Lynn M. Westphal

**Affiliations:** 1 Department of Obstetrics and Gynecology, Division of Reproductive Medicine, Stanford University, Stanford, California, United States of America; 2 Department of Obstetrics and Gynecology, Division of Reproductive Medicine, University of California at San Francisco, San Francisco, California, United States of America; Albert Einstein College of Medicine, United States of America

## Abstract

**Background:**

Disease prevalence and response to medical therapy may differ among patients of diverse ethnicities. Poor outcomes with in vitro fertilization (IVF) treatment have been previously shown in Indian women compared to Caucasian women, and some evidence suggests that poor embryo quality may be a cause for the discrepancy. In our center, only patients with the highest quality cleavage stage embryos are considered eligible for extending embryo culture to the blastocyst stage. We compared live birth rates (LBR) between Indian and Caucasian women after blastocyst transfer to investigate whether differences in IVF outcomes between these ethnicities would persist in patients who transferred similar quality embryos.

**Methodology/Principal Findings:**

In this retrospective cohort analysis, we compared IVF outcome between 145 Caucasians and 80 Indians who had a blastocyst transfer between January 1, 2005 and June 31, 2007 in our university center. Indians were younger than Caucasians by 2.7 years (34.03 vs. 36.71, P = 0.03), were more likely to have an agonist down regulation protocol (68% vs. 43%, P<0.01), and were more likely to have polycystic ovarian syndrome (PCOS), although not significant, (24% vs. 14%, P = 0.06). Sixty eight percent of Indian patients had the highest quality embryos (4AB blastocyst or better) transferred compared to 71% of the Caucasians (P = 0.2). LBR was significantly lower in the Indians compared to the Caucasians (24% vs. 41%, P<0.01) with an odds ratio of 0.63, (95%CI 0.46–0.86). Controlling for age, stimulation protocol and PCOS showed persistently lower LBR with an adjusted odds ratio of 0.56, (95%CI 0.40–0.79) in the multivariate analysis.

**Conclusions/Significance:**

Despite younger age and similar embryo quality, Indians had a significantly lower LBR than Caucasians. In this preliminary study, poor prognosis after IVF for Indian ethnicity persisted despite limiting analysis to patients with high quality embryos transferred. Further investigation into explanations for ethnic differences in reproduction is needed.

## Introduction

The epidemiology of disease and response to medical therapy can differ between ethnicities. Several disease processes have been shown to differ between patients of Indian ethnicity compared to Caucasian ethnicity. For example, populations from a South Asian Indian background have a markedly higher risk for and mortality from cardiovascular disease compared to Caucasian populations [1], [2]. Research in women's health has shown a higher rate of insulin resistance and incidence of polycystic ovarian syndrome (PCOS) in Indian women compared to Caucasian women [3].

Ethnic differences in fertility and IVF outcome have also been identified. Some European studies have found worse prognosis for women of Indian ethnicity compared to women of Caucasian ethnicity [4]–[7]. The cause behind the poor prognosis after treatment with IVF in Indians compared to Caucasians remains unclear. Two of the studies suggested that poor embryo quality in Indian patients may account for the lower pregnancy rates in this population [5], [6].

We investigated whether IVF prognosis between Indian and Caucasian ethnicities would differ in patients who transferred the highest quality embryos. The standard day of embryo transfer in our center is day 3 after oocyte retrieval; however, we consider extending the embryo culture two more days to the blastocyst stage if the embryo cohort has three or more excellent quality embryos on day 3. The extended culture allows for better embryo selection and fewer embryos to be transferred on day 5 after oocyte retrieval, but has a small risk of have no further cleavage and no embryo transfer.

We compared LBR in patients of Indian and Caucasian ethnicity after blastocyst transfer in order to investigate whether the differences in IVF outcome reported in these populations in previous studies would persist in patients with similar high quality embryos transferred.

## Results

A total of 225 patients met inclusion criteria, 64% Caucasian and 36% Indian. The Indian patients were younger than the Caucasian patients by a mean of 2.7 years (P = 0.03). Otherwise, the patients were similar in patient characteristics such as CD3 FSH, BMI, obstetric history, number of previous IVF cycles, and diagnoses for IVF (Table 1). A higher percentage (24%) of Indian patients had PCOS compared to Caucasian patients (14%), however this difference did not reach statistical significance (P = 0.06).

**Table 1:** Patient Characteristics.

Variable	Caucasian	Indian	P-value
	N = 145	N = 80	
Woman's Age	36.71±3.90 (25.94–45.95)	34.03±4.09 (25.73–41.71)	0.03
Body Mass Index	24.4±4.6 (17.5–40.1)	25.2±3.7 (16.8–35.6)	0.2
Nulligravid	43%	48%	0.3
Nulliparous	77%	76%	0.3
Prior spontaneous abortion	37%	36%	0.2
Cycle Day 3 FSĤ	6.5±2.0 (2.1–13.9)	6.2±1.8 (2.5–11.7)	0.4
Number of Previous Cycles (%)	1.1±1.3	0.8±0.8	0.2
Diminished Ovarian Reserve	10%	6%	0.09
Male Factor	36%	38%	0.2
Endometriosis	11%	15%	0.1
PCOS*	14%	24%	0.06
Tubal factor	9%	10%	0.2
Unexplained Infertility	18%	16%	0.1

ˆFSH = follicle stimulating hormone *PCOS = polycystic ovarian syndrome.

The details of the IVF cycles of each group are compared in Table 2. A higher percentage of Indian patients had the long protocol and a higher percentage of Caucasian patients had an antagonist protocol. This difference is most likely explained by the difference in average age of the two groups since our clinic typically uses the antagonist protocol in older patients with a higher chance of having a poor response to ovarian stimulation. The two groups had a similar mean total dose of gonadotropins: 3714 IU for Caucasian patients and 3106 IU for Indian patients (P = 0.2). Other aspects of the IVF cycles were similar between the two groups including mean endometrial thickness, total number of oocytes retrieved, percentage of patients using ICSI, fertilization rate, mean number of 2PN embryos, mean number of 8 cell embryos, and mean number of blastocysts.

**Table 2:** Cycle Characteristics and Response.

Variable	Caucasian	Indian	P-value
	N = 145	N = 80	
Antagonist Stimulation Protocol	48%	30%	<0.01
Long Luteal Lupron Stimulation Protocol	43%	68%	<0.01
Microdose Flare Stimulation Protocol	9%	2%	0.09
Total Dose of Gonadotropins (IU)	3714±1550	3106±1383	0.2
Endometrial Lining	10.3±1.7 (6.5–15.0)	9.9±1.8 (7.0–15.3)	0.1
Oocytes Retrieved	16.8±5.9	17.1±5.9	0.6
ICSI*	44%	43%	0.9
Fertilization Rate	69%	67%	0.7
Number of 8 Cells on Day 3	6.0±2.8	5.8±2.4	0.8
Number of Blastocysts	5.2±2.9	5.6±3.2	0.6
Embryos Transferred	2.0±0.7	1.9±0.7	0.6
Embryos transferred 4AB quality or higher	68%	71%	0.2
Number of Blastocysts Frozen	2.6±2.7	3.1±3.1	0.2

* ICSI = intracytoplasmic sperm injection.

The two groups had a similar number of embryos transferred, 2.0 in Caucasians and 1.9 in Indians (P = 0.6). The quality of embryos was similar between the two groups. The percentage of high quality blastocysts transferred, defined as 4AB or better, was 68% in the Caucasian patients and 71% in the Indian patients (P = 0.2). Our center only freezes blastocysts of 3BB quality or better and the mean number of frozen blastocysts was similar between the two groups: 2.6 for Caucasian patients and 3.1 for Indian patients (P = 0.5).

IVF cycle outcomes are reviewed in Table 3. Implantation rate was higher in the Caucasian group, although this did not reach statistical significance (39% vs. 28%, P = 0.06). The rate of positive pregnancy test was similar between the two groups (61% vs. 53%, P = 0.2). Indian patients had a significantly lower clinical pregnancy rate compared to the Caucasian patients (36% vs. 52%, P = 0.02). LBR was significantly lower in the Indian patients compared to the Caucasian patients (24% vs. 41%, P<0.01) with an odds ratio of 0.63, (95%CI 0.46–0.86). Multiple pregnancy rate was similar between the two groups. In the Caucasian patients, 17 patients delivered multiples (12%), 16 sets of twins and one triplet pregnancy. In the Indian patients, 7 patients delivered twins (9%) and no triplets were delivered.

**Table 3:** Outcomes.

Variable	Caucasian	Indian	P-value
	N = 145	N = 80	
Implantation Rate	39%	28%	0.06
Positive HCG *	61%	53%	0.2
Clinical Pregnancy*	52%	36%	0.02
SABˆ	22%	31%	0.4
Live Birth Rate*	41%	24%	0.003
Multiple Pregnancy*	12%	9%	0.5

* per embryo transfer.

ˆ per clinical pregnancy.

Controlling for age, stimulation protocol and diagnosis of PCOS showed persistently lower LBR with an adjusted odds ratio of 0.56, (95%CI 0.40–0.79) in the multivariate analysis. A diagnosis of PCOS was included in the multivariate analysis because the difference between the two groups approached statistical significance (P = 0.06) and PCOS in Indian women appears to be clinically significant in regards to fertility (see Discussion). Thus, differences seen in these variables between the two ethnicities do not account for the lower clinical pregnancy rate and LBR seen between the two groups.

## Discussion

This cohort comparison of IVF outcome after blastocyst transfer in women of different ethnicities found a significantly lower pregnancy rate and LBR in Indian women compared to Caucasian women during the same time period in our program. This worse prognosis was seen despite the fact that the Indian women were younger in age, had a similar response to ovarian stimulation, and had similar embryo quality compared to the Caucasian women.

Previous studies have shown worse prognosis for Indian women compared to Caucasian women after IVF treatment. Mahmud et al. found higher cycle cancellation rates and lower LBR in 44 Indian women compared to 88 Caucasian women matched for age and BMI despite similar doses of medication and number of oocytes retrieved [6]. Lashen et al. found a lower implantation rate (13% and 17%) and clinical pregnancy rate (16% and 23%) in 108 Indian women compared to 216 Caucasian women after IVF [5]. Although this difference did not reach statistical significance, this study did find a higher incidence of polycystic appearing ovaries in the Indian group.

Several theories exist regarding the possible cause for subfertility in the Indian population compared to Caucasians seen in these studies. One common theory is a higher incidence of PCOS in Indian women. Several studies recognize a higher incidence of PCOS in Indian women compared to other ethnicities [3], [8], [9]. PCOS is a complex, multifaceted metabolic disorder associated with insulin resistance, hyperandrogenism, and ovulation dysfunction leading to subfertility. Women of Indian ethnicity seem to be more sensitive to the metabolic effects of this disorder and Rodin et al. showed increased insulin resistance in Indian women compared to Caucasian women at a similar BMI [3]. Palep-Singh et al. found that Caucasian patients with PCOS had a 2.5 times (95% CI: 1.25–5) higher chance of an ongoing clinical pregnancy per cycle compared to their Indian counterparts [10]. In our study, a higher percentage of Indian women had PCOS compared to the Caucasian group (24% vs. 14%), however this difference did not reach statistical significance (P = 0.06).

Some research suggests that a high incidence of genital tuberculosis (TB) in the Indian population may explain subfertility in that population [11]. In a study of 300 women in Bombay, India, being evaluated for tubal factor as a cause of infertility, 39% were found to have genital TB diagnosed by endometrial biopsy, serologic testing, or pathology from laparoscopic specimens. Dam et al. suggest that treatment for latent TB should be considered in Indian patients presenting with unexplained infertility and repeated IVF failure [12]. None of the patients in our study had been evaluated for genital TB by serologic testing or tissue culture, however, none of the patients had uterine scarring based on evaluation by hysteroscopy or hysterosalpigogram. Physicians treating infertile women who have lived in TB endemic areas may consider its evaluation under certain circumstances.

Some evidence suggests differences in rates of endometriosis associated infertility among different ethnicities [13]–[14]. Miyazawa first noted ethnicity as a risk factor for endometriosis when he noted a higher incidence of the disease in Japanese women compared to Caucasians [15]. Arumgam et al. found a higher incidence of endometriosis in patients from Malaysia undergoing diagnostic laparoscopy for infertility compared to Caucasian women in the United Kingdom undergoing the same procedure [16]. Clinical teaching suggests a higher incidence of endometriosis in Indian women compared to Caucasian women, however, a thorough literature review found no evidence of this association. Endometriosis as an indication for in vitro fertilization did not differ in Indians and Caucasians in our study (Table 1).

Our study has some limitations. We state that the groups had similar embryo quality based on current standards of embryo evaluation: visualization of cell number, degree of fragmentation. Assessing embryo quality in this manner does not account for genetic and epigenetic differences in embryos that may be associated with IVF outcome. In addition, we only have ethnic information on the female patient and we do not know the country of birth or the length of time living in the U.S. Future studies evaluating ethnicity and fertility should include male partner ethnicity and more detailed information on time in U.S. since environmental exposure may play a key role in fertility and prognosis for treatment.

To our knowledge this is the largest comparison of IVF outcome between Indian and Caucasian patients to date, and the only study to include live birth rate after blastocyst transfer as the primary outcome. It is striking that prognosis of live birth is lower in Indian women despite their being younger and having similar embryo quality compared to the Caucasian population.

Our findings are very interesting but preliminary and we are hesitant to suggest an explanation for the worse prognosis of Indian women to Caucasian after treatment with IVF. Although we attempt to control for embryo quality by only comparing patients with a blastocyst transfer, we are limited by the current assessment of embryo quality. Future investigations may reveal molecular differences between embryos from women of different ethnicities. A difference in implantation environment in Indian women, due to PCOS, tuberculosis, and endometriosis, has been suggested in previous studies, but our patients were not universally screened for these conditions. Of the patients who were known to have PCOS or endometriosis in our population, the incidence of these conditions did not differ between the two ethnicities.

Future epidemiologic studies on ethnicity in IVF outcome should be prospective and include more information on male ethnicity, exposure to tuberculosis, thorough evaluation for PCOS, place of birth and length of time living in different countries. Further work should include investigation of metabolic, endocrine, and immunologic differences between ethnicities which may guide future treatment options of subfertility.

## Materials and Methods

We conducted a retrospective review of all fresh, non-donor IVF cycles resulting in blastocyst transfers from January 1, 2005, to June 31, 2007, in our university IVF center. A questionnaire, completed by all patients before their first visit to our clinic, included a write-in response for ethnicity. Patients who answered Caucasian or white in this section were included as Caucasian ethnicity and patients who wrote in Indian were considered Indian ethnicity for the purposes of this study. Any blastocyst transfers with preimplantation genetic diagnosis were excluded. If patients had multiple IVF cycles resulting in blastocyst transfer during this time period, only the first cycle was included.

Institutional review board approval from Stanford University's Administrative Panels on Human Subjects in Medical Research was obtained to conduct this study. Data was gathered anonymously so individual patient consent was not obtained. The intuitional review board approved the waiver of consent for this study.

Patients received one of three standard ovarian stimulation protocols: microdose lupron (flare), antagonist, or luteal down regulation (long). Details of these protocols have been described previously [17]. Younger, good prognosis patients typically will follow the luteal down regulation protocol and older patients or those with poor prognosis will follow either the microdose lupron or antagonist protocols. Ultrasound monitoring of follicular growth was performed after four days of gonadotropin stimulation and approximately every other day as indicated. A dose of 10,000 units of human chorionic gonadotropin (hCG) was administered when at least one follicle reached an average diameter of 17 mm or greater. Oocyte retrieval was performed by transvaginal ultrasound guidance 35 hours after hCG administration.

Fertilization of the oocytes was achieved with standard insemination or intracytoplasmic sperm injection (ICSI) depending on semen parameters or history of low fertilization. The oocytes were examined for fertilization status 16–18 hours after IVF or ICSI. The zygotes with two pronuclei were cultured for 24–48 hours in Sage Cleavage Medium (Cooper Surgical, Inc, Trumbull, CT) with 10% Serum Protein Substitute (SPS, Irvine Scientific, Santa Ana, CA).

A single team of experienced embryologists evaluated the embryos on day 3 after oocyte retrieval, 68 to 72 hours after oocyte harvest. Embryos were examined for cleavage (cell number) and grade, which includes cytoplastmic fragmentation. Embryos were graded as follows on day 3: Grade 1, blastomeres have equal size and no cytoplasmic fragmentation; Grade 2, blastomeres have equal size and minor cytoplasmic fragmentation involving <10% of the embryo; Grade 3, blastomeres have unequal size and fragmentation involving 10–20% of the embryo; Grade 4, blastomeres have equal or unequal size, and moderate to significant cytoplasmic fragmentation covering 20–50% of the embryo; and Grade 5, few blastomeres and severe fragmentation covering ≥50% of the embryo [18].

If the embryo cohort had 3 or more 8 cell embryos grade 1 or 2 on day 3 after oocyte retrieval, then embryos were cultured to the blastocyst stage in Blastocyst Medium (Irvine Scientific, Santa Ana, CA) with 10% serum substitute supplement. Blastocysts were classified with the grading system described previously [19]. We defined good quality blastocysts based on a study by della Ragione et al. showing that 4AA, 4AB, or better blastocysts have better implantation potential and pregnancy outcome compared to poorer quality blastocysts after a single embryo transfer [20]. Any expanding, expanded, and hatching blastocysts with good inner cell mass and trophectoderm epithelium (3BB or better) were frozen on day 5 or day 6 with protocols described elsewhere [21]. Embryos which either arrested or were of worse quality than 3BB were discarded or donated to research.

Transabdominal ultrasound-guided embryo transfer on day 5 or 6 after oocyte retrieval was performed using a Tefcat or Echotip Softpass catheter (Cook Ob/GYN, Spencer, IN). All patients received progesterone supplementation with vaginal suppositories (200 mg three times a day) starting on the evening after oocyte retrieval.

Data collected included patient characteristics: age, obstetric history, body mass index (BMI), cycle day 3 FSH, number of previous IVF cycles, and indication for IVF. PCOS was defined by the Rotterdam criteria [22]. Data collected on IVF cycles collected included ovarian stimulation protocol, amount of gonadotropins used, endometrial lining thickness, number of oocytes retrieved, fertilization method, fertilization rate, number of 8 cell embryos on Day 3, number of blastocysts, number of embryos transferred, and number of blastocysts frozen. The primary outcome measured was LBR and secondary outcomes included were implantation rate, positive pregnancy test, clinical pregnancy, spontaneous pregnancy loss, and multiple pregnancy rate.

Implantation rate was defined as number of gestational sacs seen on transvaginal ultrasound at 6 weeks gestation per number of embryos transferred. A pregnancy test was considered positive if the beta-hCG level was >5 mIU/mL 10–11 days after embryo transfer. Clinical pregnancy was defined as gestational sac seen on transvaginal sonogram at 6–7 weeks gestational age. Spontaneous pregnancy loss was defined as loss of pregnancy after gestational sac seen on transvaginal ultrasound at 6 weeks gestation. Live birth data were collected by contacting patients with a 100% follow up rate.

Continuous variables were examined for normality; if normally distributed, compared with a Student's t test, and if not normally distributed, compared with a nonparametric test. Dichotomous variables were compared with a chi squared test. All statistical tests were two-tailed and used an alpha of 0.05. Univaritate analyses were performed to determine baseline characteristic and cycle parameter differences between the Indian population and the Caucasian population. To estimate the independent contribution of Indian ethnicity on treatment outcomes, a multivariable logistic regression analysis was performed. Potential confounders found to be statistically significant in univariate analyses (Tables 1 and 2) and others generally regarded as clinically significant were included in the model. The final model included age, diagnosis of PCOS and protocol. Data were analyzed using STATA, version 9.0 (College Station, TX), and SAS, version 8.02 (Cary, NC).
